# Supplementary material for: Circuit implementation of a four-dimensional topological insulator
Source: Nat Commun. 2020 May 12;11:2356. doi: 10.1038/s41467-020-15940-3 (PMC7217906; doi:10.1038/s41467-020-15940-3)
Supplement: Supplementary file 1 — Supplementary Information [file 41467_2020_15940_MOESM1_ESM.pdf]

Supplementary Information for:
Circuit Implementation of a Four-Dimensional Topological Insulator

Wang et al.

SUPPLEMENTARY NOTE 1: TIGHT-BINDING MODEL

The lattice has four sites denoted by (A, B, C, D). With the distance between nearest-neighbor lattice sites normalized to 1, the relative coordinates of sites A, B, C, D in each unit cell are respectively $(0,0,0,0)$, $(1,0,1,0)$, $(1,0,0,0)$ and $(0,0,1,0)$, and the lattice vectors are $\mathbf{R}_1 = (1,-1,0,0)$, $\mathbf{R}_2 = (1,1,0,0)$, $\mathbf{R}_3 = (0,0,1,-1)$, and $\mathbf{R}_4 = (0,0,1,1)$.

The full real-space tight-binding Hamiltonian is written in second-quantized notation as [arXiv:1806.05263]:

$$
\begin{aligned}
H &= H_x + H_y + H_z + H_w + H_1 + H_2,\\
H_x &= J\sum_{mnjl}\Big(c^\dagger_{mnjl}a_{mnjl} + a^\dagger_{m+1,n+1,jl}c_{mnjl} - b^\dagger_{mnjl}d_{mnjl} - d^\dagger_{m+1,n+1,jl}b_{mnjl} + \text{h.c.}\Big)\\
H_y &= J\sum_{mnjl}\Big(c^\dagger_{m-1,njl}a_{mnjl} - b^\dagger_{m-1,njl}d_{mnjl} + \text{h.c}\Big)\\
H_z &= J\sum_{mnjl}\Big(d^\dagger_{mnjl}a_{mnjl} + a^\dagger_{mnj+1,l+1}d_{mnjl} + b^\dagger_{mnjl}c_{mnjl} + c^\dagger_{mnj+1,l+1}b_{mnjl} + \text{h.c.}\Big)\\
H_w &= J\sum_{mnjl}\Big(d^\dagger_{mnj-1,l}a_{mnjl} + b^\dagger_{mnj-1,l}c_{mnjl} + \text{h.c}\Big)\\
H_1 &= m\sum_{mnjl}\Big(a^\dagger_{mnjl}a_{mnjl} + b^\dagger_{mnjl}b_{mnjl} - c^\dagger_{mnjl}c_{mnjl} - d^\dagger_{mnjl}d_{mnjl}\Big)\\
H_2 &= J'\sum_{mnjl}\Big(a^\dagger_{m+1,n+1,j+1,l+1,}a_{mnjl} + b^\dagger_{m+1,n+1,j+1,l+1,}b_{mnjl}\\
&\qquad\qquad - c^\dagger_{m+1,n+1,j+1,l+1,}c_{mnjl} - d^\dagger_{m+1,n+1,j+1,l+1,}d_{mnjl} + \text{h.c}\Big)\\
&\quad + J''\sum_{mnjl}\Big(a^\dagger_{m+1,n+1,j-1,l-1,}a_{mnjl} + b^\dagger_{m+1,n+1,j-1,l-1,}b_{mnjl}\\
&\qquad\qquad - c^\dagger_{m+1,n+1,j-1,l-1,}c_{mnjl} - d^\dagger_{m+1,n+1,j-1,l-1,}d_{mnjl} + \text{h.c}\Big).
\end{aligned}
$$

Here, the (m, n, j, l) subscripts index each unit cell in terms of elementary lattice vectors, and the α_{mnjl} ($\alpha^\dagger_{mnjl}$) operators annihilate (create) a particle on site $\alpha \in \{a, b, c, d\}$.

By Fourier transforming the operators and choosing $J = 1$ and $J' = -J'' = 2$, we obtain the k-space Hamiltonian

$$
\begin{aligned}
H(\mathbf{k}) = (2\cos k_x + \cos k_y)\Gamma_1 + \sin k_y\Gamma_2 + (2\cos k_z + \cos k_w)\Gamma_3\\
+ \sin k_w\Gamma_4 + \big[m + 4\cos(2k_x + 2k_z) - 4\cos(2k_x - 2k_z)\big]\Gamma_5, \quad (1)
\end{aligned}
$$

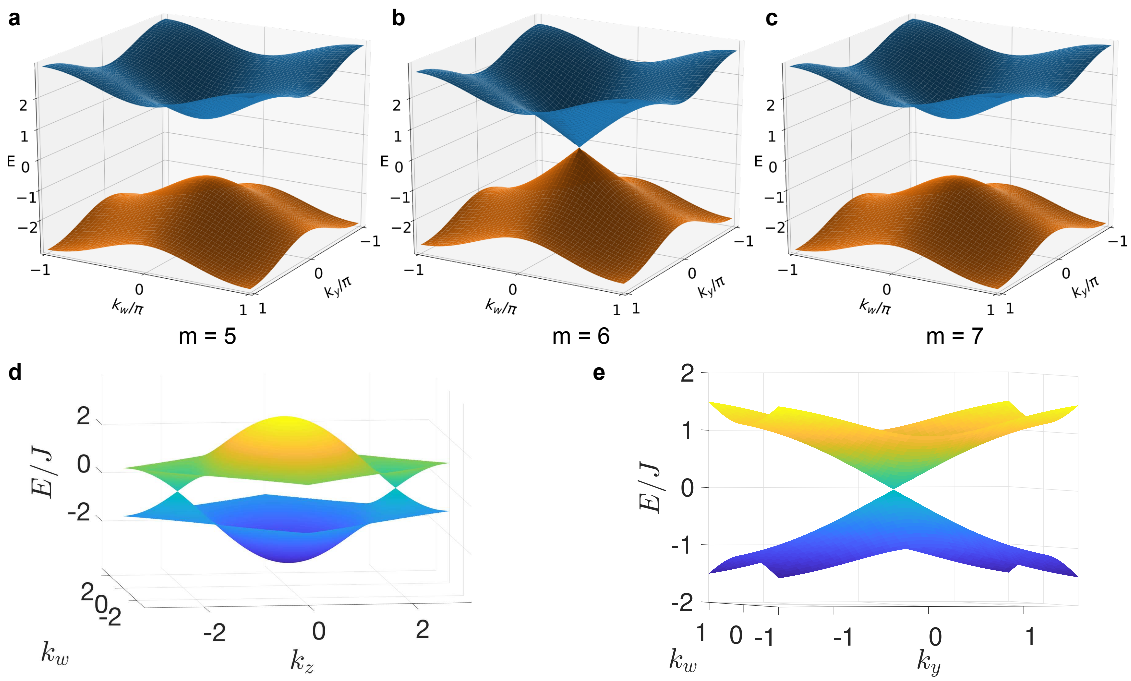

Supplementary Figure 1. **Bulk and surface dispersion relations.** **a**–**c** Bulk dispersion of the tight-binding model in the k_y-k_w plane, with $k_x = k_z = 2\pi/3$, for $m = 5, 6, 7$. **d**–**e** Surface dispersion for a lattice with $m = 0$ and truncated to $N_x = 30$ unit cells (with open boundary conditions) in the x direction, plotted in the k_w-k_z plane for $k_y = 0$ (**d**), and in the k_w-k_y plane for $k_z = 2\pi/3$ (**e**).

where Γ are the Dirac matrices

$$\Gamma_1 = \begin{pmatrix} 0 & 0 & 1 & 0 \\ 0 & 0 & 0 & -1 \\ 1 & 0 & 0 & 0 \\ 0 & -1 & 0 & 0 \end{pmatrix}, \Gamma_2 = \begin{pmatrix} 0 & 0 & -i & 0 \\ 0 & 0 & 0 & -i \\ i & 0 & 0 & 0 \\ 0 & i & 0 & 0 \end{pmatrix}, \Gamma_3 = \begin{pmatrix} 0 & 0 & 0 & 1 \\ 0 & 0 & 1 & 0 \\ 0 & 1 & 0 & 0 \\ 1 & 0 & 0 & 0 \end{pmatrix}, \Gamma_4 = \begin{pmatrix} 0 & 0 & 0 & -i \\ 0 & 0 & i & 0 \\ 0 & -i & 0 & 0 \\ i & 0 & 0 & 0 \end{pmatrix}, \Gamma_5 = \begin{pmatrix} 1 & 0 & 0 & 0 \\ 0 & 1 & 0 & 0 \\ 0 & 0 & -1 & 0 \\ 0 & 0 & 0 & -1 \end{pmatrix}.$$

For $k_x = k_z = 2\pi/3$, Fig. 1 shows the $k_y - k_w$ dispersion near one of the four Dirac cones. The gap closes at $m = 6$ with the Dirac point lying at $k_y = k_w = 0$.

When the lattice is truncated, surface states appear along the surface in the topologically nontrivial phase ($m \leq 6$), as shown in Fig. 1**d**–**e**. In this case, the truncation occurs along the x direction, and the surface state cones are centered at $k_y = k_w = 0$, $k_z = \pm 2\pi/3$.

SUPPLEMENTARY NOTE 2: CIRCUIT DESIGN DETAILS

Here, we provide additional details on the mapping between the electric circuits and tight-binding Hamiltonians. Let I_i be the external current injected into node i, V_j the voltage (relative to ground) on node j, and D_{ij} the conductance between nodes i and j for $i \neq j$. Moreover, let the conductance between node i and ground be

$$D_{ii}^{(g)} = -D_{ii} + D'_{ii}. \tag{2}$$

By Kirchhoff's laws,

$$\begin{aligned} I_i &= D_{ii}^{(g)} V_i + \sum_j D_{ij}(V_i - V_j) && (3) \\ &= \sum_j \left[-D_{ij} + \left(-D_{ii} + D'_{ii} + \sum_k D_{ik} \right) \delta_{ij} \right] V_j && (4) \\ &= \sum_j \left[-D_{ij} + \left(D'_{ii} + \sum_{k\neq i} D_{ik} \right) \delta_{ij} \right] V_j. && (5) \end{aligned}$$

Note that in Eq. (3), the sum can be taken either over all j, or equivalently over $j \neq i$. We now adjust D'_{ii} so that, at a reference working frequency f_0,

$$D'_{ii}(f_0) + \sum_{j\neq i} D_{ij}(f_0) = i\alpha E \tag{6}$$

for each node i, with some constant α and target energy E. At $f = f_0$, Eq. (5) then becomes

$$\begin{aligned} I_i(f_0) &= -i\alpha \sum_j \Big[H_{ij}(f_0) - E\,\delta_{ij} \Big] V_j(f_0), && (7) \\ D_{ij}(f) &\equiv i\alpha H_{ij}(f). && (8) \end{aligned}$$

We require $H_{ij}(f_0)$ to match the target tight-binding Hamiltonian, which has parameters $J = 1$, $J' = -J'' = 2$. For real α, positive (negative) real values of H_{ij} correspond to capacitances (inductances). As described in the main text, by choosing α and f_0 we can assign the following circuit elements to the lattice model's hopping terms:

$$\begin{array}{llll} C_0 & \leftrightarrow \;\; J & = 1 & \text{(positive NN hopping)} \\ C' = 2C_0 & \leftrightarrow \;\; J' & = 2 & \text{(positive long range hopping)} \\ L_0 & \leftrightarrow -J & = -1 & \text{(negative NN hopping)} \\ L' = L_0/2 & \leftrightarrow \;\; J'' & = -2 & \text{(negative long range hopping)} \end{array} \tag{9}$$

where

$$2\pi f_0 = 1/\sqrt{L_0 C_0}, \qquad \alpha = 2\pi f_0 C_0. \tag{10}$$

For each node, we determine the grounding conductance required to satisfy Eq. (6). Suppose node i is connected to other nodes by p_i type-C_0 capacitors, q_i type-L_0 inductors, p_i' type-C' capacitors, and q_i' type-L' inductors (these connections depend on which sublattice the node lies on, and whether it lies in the bulk or on the surface). Then

$$\begin{aligned}\sum_{j\neq i} D_{ij}(f) &= 2\pi i p_i f C_0 + \frac{q_i}{2\pi i f L_0} + 2\pi i p_i' f C' + \frac{q_i'}{2\pi i f L'} \\ &= 2\pi i f\, C_0 \left(p_i + 2p_i' - (q_i + 2q_i')\,\frac{f_0^2}{f^2}\right).\end{aligned} \tag{11}$$

Taking $f = f_0$ and plugging into Eq. (6) gives

$$\begin{aligned}D_{ii}'(f_0) &= i\alpha E - \sum_{j\neq i} D_{ij}(f_0) \\ &= 2\pi i f_0 C_0 \big(E - p_i - 2p_i' + q_i + 2q_i'\big).\end{aligned} \tag{12}$$

The on-site mass term is $H_{ii}(f_0) = \pm m$, depending on whether the node is on the A,B or C,D sublattices. Hence, the grounding conductance must satisfy

$$\begin{aligned}D_{ii}^{(g)}(f_0) &= -D_{ii}(f_0) + D_{ii}'(f_0) \\ &= 2\pi i f_0 C_0 \big(E \mp m - p_i - 2p_i' + q_i + 2q_i'\big).\end{aligned} \tag{13}$$

To achieve this in the experiment, we connect each node i to ground with $6 - p_i$ type-C_0 capacitors, $3 - q_i$ type-L_0 inductors, $4 - p_i'$ type-C' capacitors, and $4 - q_i'$ type-L' inductors. Additionally, (i) we connect each node to ground by an extra inductor L_g, and (ii) if node i belongs to sublattice C or D, we connect it to ground by an extra capacitor $C_m = 2mC_0$. As a result, the grounding conductance of node i at an arbitrary frequency f is

$$D_{ii}^{(g)} = 2\pi i(6 - p_i) f C_0 + \frac{(3 - q_i)}{2\pi i f L_0} + 2\pi i(4 - p_i') f C' + \frac{(4 - q_i')}{2\pi i f L'} + \frac{1}{2\pi i f L_g} + 2\pi i(m \mp m) f C_0 \tag{14}$$

where $\mp$ refers to sublattice A,B or C,D respectively. At $f = f_0$, this satisfies Eq. (13) if we pick

$$\frac{L_0}{L_g} = 3 + m - E. \tag{15}$$

Hence,

$$D_{ii}^{(g)}(f) = 2\pi i f C_0 \left[14 + m \mp m - p_i - 2p_i' + \Big(E - 14 - m + q_i + 2q_i' \Big) \frac{f_0^2}{f^2} \right]. \qquad (16)$$

Returning to Eq. (5), define the quantity in the parentheses—which gives rise to the E term in Eq. (7)—as

$$i\alpha\, \mathcal{E}_i(f) = D_{ii}'(f) + \sum_{k \neq i} D_{ik}(f) \qquad (17)$$

$$= D_{ii}^{(g)}(f) + D_{ii}(f) + \sum_{j \neq i} D_{ij}(f) \quad \xrightarrow{f \to f_0} \quad i\alpha E. \qquad (18)$$

Eq. (7) then generalises to

$$I_i(f) = -i\alpha \sum_j \Big[H_{ij}(f) - \mathcal{E}_i(f)\, \delta_{ij} \Big] V_j(f). \qquad (19)$$

Now observe that in Eq. (18), the first term $D_{ii}^{(g)}(f)$ is defined by Eq. (16) for any f, and the third term is likewise defined by Eq. (11) for any f. However, $D_{ii}(f)$ is defined only at $f = f_0$. This turns out not to be a problem for our system of equations, since this term is exactly cancelled by the Hamiltonian term in Eq. (19), which possesses the same ambiguity. We are therefore free to give $D_{ii}(f)$ any frequency dependence, consistent with its value at f_0 (i.e., $D_{ii}(f_0) = i\alpha H_{ii}(f_0) = \pm i\alpha m$). A convenient choice is

$$D_{ii}(f) = i\alpha E - D_{ii}^{(g)}(f) - \sum_{j \neq i} D_{ij}(f) \quad \xrightarrow{f \to f_0} \quad i\alpha H_{ii}(f_0) \qquad (20)$$

$$\Rightarrow \quad i\alpha\, \mathcal{E}_i(f) = i\alpha E \quad \text{for all } \; i, f. \qquad (21)$$

With this choice, $\mathcal{E}_i(f)$ becomes i-independent, and Eq. (19) simplifies to

$$I_i(f) = -i\alpha \sum_j \Big[H_{ij}(f) - E\, \delta_{ij} \Big] V_j(f). \qquad (22)$$

This can be interpreted as a family of response equations with an f-dependent Hamiltonian and fixed energy E. For general f, the Hamiltonian's hopping terms are determined by the circuit elements summarised in Eq. (9), and its on-site mass term is determined by Eq. (20); for $f = f_0$, it reduces to the target Hamiltonian.

Suppose E is in a topological gap of the target Hamiltonian, so that topological surface states exist at frequency f_0. As we vary f away from f_0, the Hamiltonian varies smoothly,

deviating from the form of the target Hamiltonian (e.g., the positive and negative nearest neighbor hoppings become unequal in magnitude). Throughout this variation, so long as E lies in a gap, the topological properties are unchanged and the topological surface states continue to exist. Thus, the f-dependent response of the circuit behaves like a bandstructure.

For small m, the circuit exhibits a finite-width topological bandgap in f-space. Tuning m closes this bandgap, and causes the surface states to disappear.

SUPPLEMENTARY NOTE 3: FURTHER CIRCUIT SIMULATION RESULTS

Supplementary Figure 2 shows the simulated LDOS profile for $E = 0, m = 0, 4, 8$ and $E = 1, m = 0$ (using 5Ω resistances), corresponding to the experimental results shown in Fig. 2**c**–**f** of the main text. Disorder is not included in these simulations.

Supplementary Figure 3 shows how the simulated frequency-dependent LDOS measure is affected by circuit resistance. The upper row shows the outcomes for realistic resistances (matching Fig. 4 and Fig. 3 of the main text). The lower row shows the results with much lower resistance (0.5Ω for all capacitors and inductors).

From these results, we see that the main effect of the circuit resistances is to smooth out the frequency dependence of the LDOS measure. The signatures of the topological surface states and bulk bandgap are present in either case.

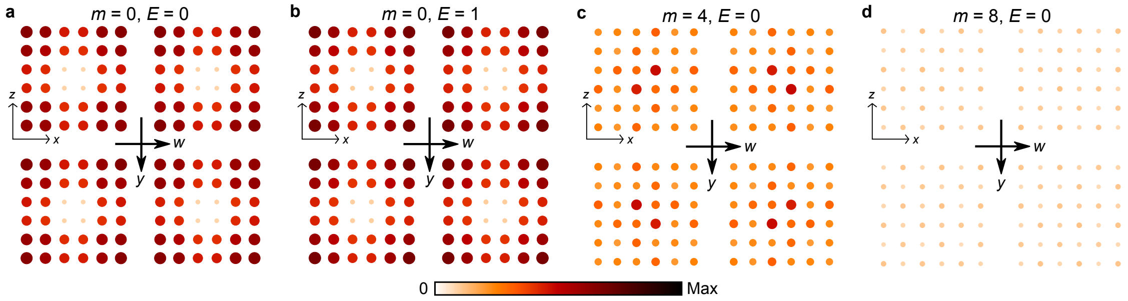

Supplementary Figure 2. **Simulated LDOS distributions**. The lattice parameters are $E = 1, m = 0$ and $E = 0, m = 0, 4, 8$; compare to Fig. 2**c**–**f** of the main text.

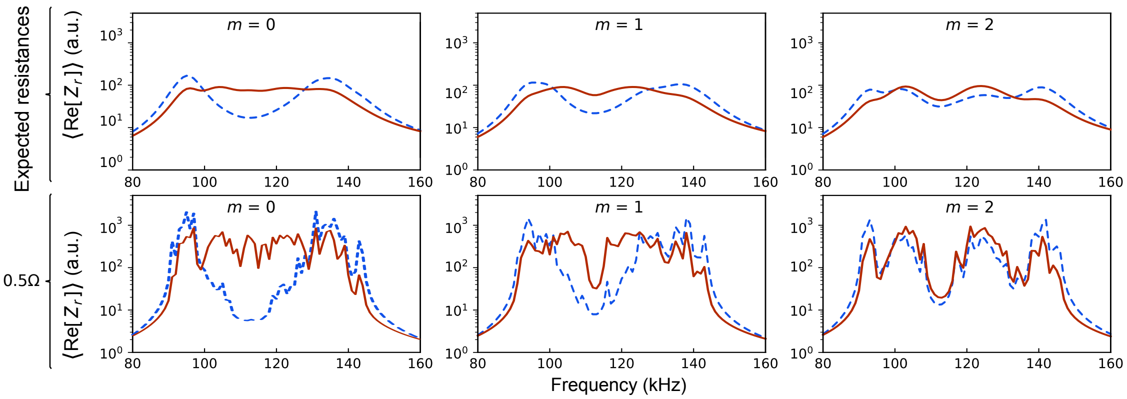

Supplementary Figure 3. **Simulated frequency-dependent LDOS measure at different resistances.** Upper row: manufacturer-provided resistances, corresponding to the first row of Fig. 4**b**. Lower row: 0.5Ω in all capacitors and inductors.

SUPLEMENTARY NOTE 4: FREQUENCY RESPONSE FOR DIFFERENT MASS PARAMETERS

Supplementary Figure 4 shows the frequency dependence of the LDOS measure $\mathrm{Re}[Z_r]$, averaged over on surface and bulk sites, for $m = 0, 1, \ldots, 8$. In Fig. 3**b**–**e** and **g**–**j** of the main text, only a few values of m were shown for brevity. The experimental results are shown in Fig. 4**a**, and the corresponding simulation results are shown in Fig. 4**b**. The discrepancies between experimental and simulation results can be explained by the disorder in the experimental samples: according to the manufacturer data sheets, individual capacitors and inductors have 10% tolerance in the stated capacitances and inductances; moreover, as discussed in Supplementary Note 4, there are variations in the resistances of the individual capacitors, inductors, and interconnects.

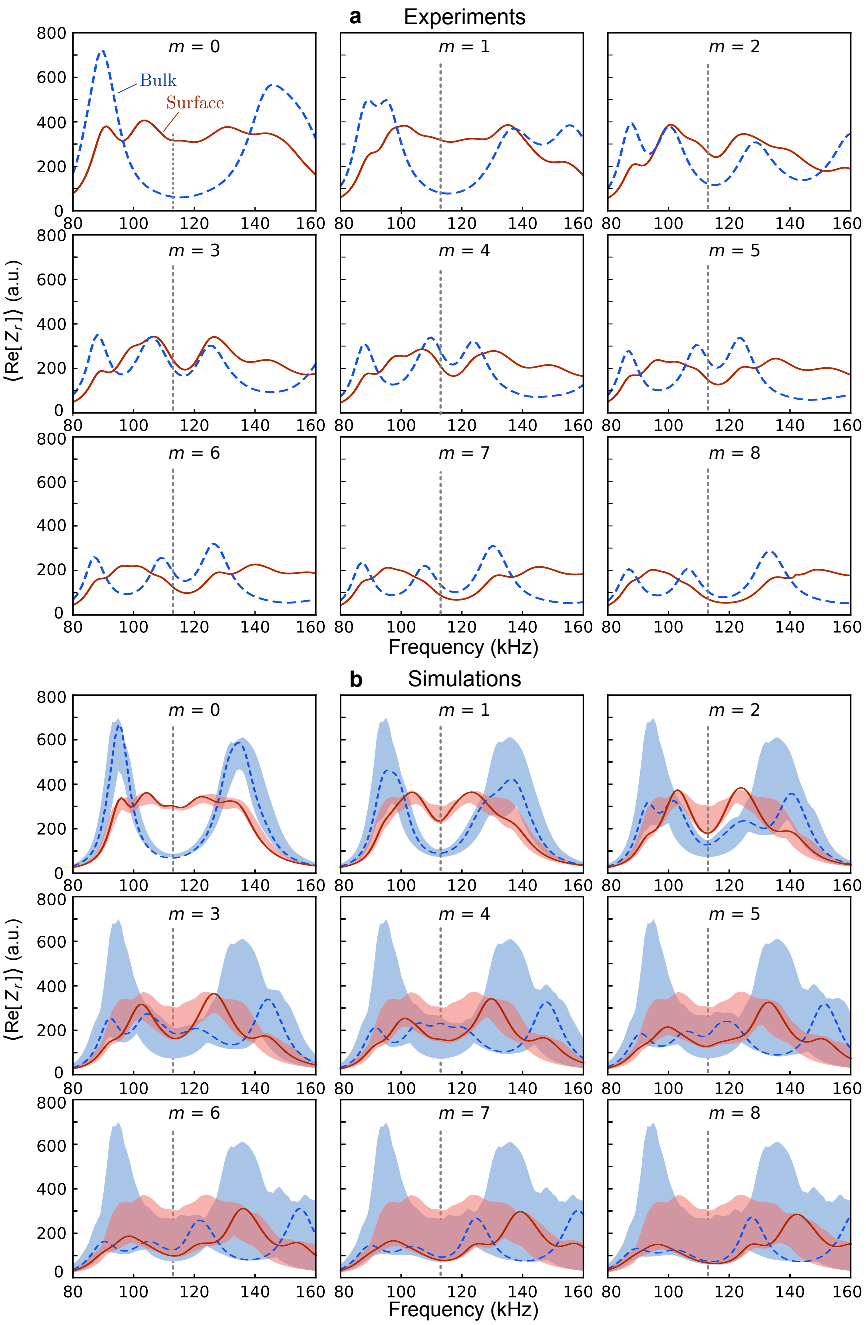

Supplementary Figure 4. **Experimental and simulated frequency responses of the circuits.** **a** Measured frequency dependence of $\mathrm{Re}[Z_r]$ averaged over surface and bulk sites, for $m = 0, 1, \ldots, 8$. The measurements are taken over sites in the 2D plane $(y, w) = (1, 0)$, and f_0 is indicated by the vertical dotted lines. **b** The corresponding circuit simulation results, with solid curves and dashes assuming no disorder in the circuit components, and red and blue areas indicating the range of impedances with 10% variation in individual capacitances and inductances, over 50 disorder realisations.
